# Abundant expression of ferroptosis-related SAT1 is related to unfavorable outcome and immune cell infiltration in low-grade glioma

**DOI:** 10.1186/s12885-022-09313-w

**Published:** 2022-02-28

**Authors:** Yanhua Mou, Lu Zhang, Zhantao Liu, Xiujun Song

**Affiliations:** 1 Department of Oncology, Xiangyang Central Hospital, Affiliated Hospital of Hubei University of Arts and Science, Xiangyang, 441021 China; 2 Department of Oncology, First Affiliated Hospital of Anhui Medical University, Hefei, 230022 China

**Keywords:** Ferroptosis, SAT1, Low-grade glioma, Pan-cancer, Immune cells, Immune checkpoint, Drug resistance

## Abstract

**Background:**

Low-grade glioma (LGG) is susceptible to ferroptosis, which is involved in TMZ resistance. Ferroptosis induction can enhance the sensitivity to TMZ and synergistically kill glioma cells. T cell-promoted tumor ferroptosis is a vital anti-tumor mechanism of immune checkpoint inhibitors. The SAT1 activation is closely related to ferroptosis upon ROS induction due to the upregulation of arachidonate 15-lipoxygenase (ALOX15) expression.

**Methods:**

The expression of SAT1 in pan-cancer and corresponding normal tissue from the TCGA data portal was primarily explored. The landscape of SAT1 and immune cell infiltration and their corresponding gene marker sets in different tissues were further explored. Additionally, we evaluated the relationships between SAT1 and the clinicopathologic parameters of LGG, and the disease-specific survival (DSS), progression-free interval (PFI), and overall survival (OS) were also assessed using KM survival curves and multivariate analysis in LGG. Meanwhile, the Gene Set Enrichment Analysis (GSEA) was also implemented to determine the potential effect of the SAT1 gene in LGG. Furthermore, the predictive power of SAT1 was validated using an independent LGG cohort from the Chinese Glioma Genome Atlas (CGGA) data.

**Results:**

In general, the expression of SAT1 is different between most tumors and their adjacent normal tissues. The results demonstrated that SAT1 expression is positively associated with TMB in LGG, BRCA, and THYM. The results displayed that the expression level of SAT1 is obviously correlated with the level of infiltrating macrophages and CD8 + T cells, and the levels of most immune gene sets were associated with the SAT1 expression in LGG. Interestingly, univariate and multivariate models significantly indicated that the OS and PFI of patients with LGG with high SAT1 levels were poorer than those with low SAT1 expression in the TCGA LGG cohort. GSEA showed that SAT1 was involved in immune regulation and multiple signaling pathways. Finally, our analysis demonstrated that SAT1 was closely associated with IDH mutation, 1p19q codeletion, chemoradiotherapy resistance and disease recurrence.

**Conclusions:**

Abundant expression of SAT1 was related to poor disease prognosis and abundant immune cell infiltration in LGG.

**Supplementary Information:**

The online version contains supplementary material available at 10.1186/s12885-022-09313-w.

## Background

Polyamines are ubiquitous amino acid-derived polycationic amines that are implicated in the major regulators of cellular growth, differentiation, and proliferation in eukaryotic cells [1]**.** Polyamine levels are tightly regulated and controlled through uptake, synthesis, transport and catabolism to maintain homeostatic. Spermidine/Spermine N1-acetyltransferase 1(SAT1) is an important global rate-limiting polyamine catabolic enzyme that catalyzes the acetylation of spermine and spermidine to N1-acetylspermine and N1-acetylspermidine respectively [2]. The SAT1 gene, located on the X chromosome (locus Xp22.3), produces two transcript variants: spermidine/spermine N-1 acetyltransferase (SSAT1), a 1085-bp mRNA, which is a key metabolic regulator in the catabolism of polyamines. SSATX, a 1195-bp novel alternative splicing variant that does not show coding potential, but can apparently modulate the activity of SSAT1 [3]**.**

The SAT1 levels are normally low, but its activity is broadly induced via a variety of numerous stimuli, including drugs (nonsteroidal anti-inflammatory drugs, 5-fluorouracil, and cisplatins), stress pathways (such as ischemia–reperfusion injury), cytokines, hormones, natural products (gossypol, resveratrol, 7-β-hydroxycholesterol, and interferons), toxins, and cachexia. Since polyamines play crucial roles in normal growth and potassium channel regulation, SAT1 is a critical enzyme in these processes [4]. Increased SSAT activity has been implicated in the damage of multiple tissues associated with pathological conditions, including kidney failure, pancreatic cell death, inflammation, diabetes and obesity, stroke, changes in bebehavior, hair loss, and keratosis follicularis spinulosa decalvans [5]. Additionally, SAT1 knockdown promoted the cancer cell proliferation, migration, and invasion, especially in melanoma [6–8].

The p53, the guardian of the genome, not only participates in the control of cell division and survival under various stresses, but also regulates cell apoptosis, autophagy and ferroptosis [9]. The SAT1 gene is a transcriptional target of p53. Previous studies have provided strong evidence to suggest that the polyamine depletion induced by AdSAT1 (an adenovirus encoding the key polyamine catabolic enzyme SAT1) transduction triggers apoptosis via the intrinsic pathway induced by mitochondrial distortions [10]. Surprisingly, Qu et al. confirmed that SAT1 is involved in the regulation of the p53-mediated reactive oxygen species (ROS) response and ferroptosis which is an iron-dependent nonapoptotic death form that can be caused by restriction of cystine uptake, a decrease in glutathione synthesis, and accumulation of lipid ROS [11]. Data from several recent researches have emphasized that iron depletion upregulates the expression of SAT1 across different cell-types.

Gliomas are the commonest primary malignant tumours of the central nervous system (CNS), accounting for 80% of malignant brain tumors. The WHO classification scheme divides glioma into four grades. The LGG constitutes 20% of all gliomas, and its main pathological types include oligodendroglioma, oligoastrocytoma, and astrocytoma. The current standard treatment for LGG is aggressive surgical operation and postoperative chemotherapy and radiotherapy [12]. In recent years, with the development of immunotherapy, targeted therapy and tumor electric field therapy, active comprehensive treatment has improved; however, the 10-year survival rate of cases is still less than 50% and more than half of LGG patients develop therapy-resistant advanced aggressive glioma over time, with depressing prognosis [13]. Previous studies have suggested that ferroptosis is involved in the occurrence and prognosis of glioma. SAT1 is involved in the regulation of ferroptosis, tumor proliferation, migration, and invasion. However, the relationship between SAT1 and LGG has not been profoundly explored before. Hence, the present study explored the correlation between SAT1 and LGG prognosis through bioinformatics analysis, which revealed the potential biological functions.

## Methods

### Data source

In the initial dataset mining, all RNA sequencing data of 33 types of cancer and normal tissue control samples from TCGA were obtained from the UCSC Xena browser (https://xenabrowser.net/) [14]. The cBioPortal for Cancer Genomics (http://www.cbioportal.org/) is a public resource for the interactive exploration of tumor genomics data [15]. Clinical information regarding patients with the 33 types of cancer downloaded from the publicly available cBioPortal online website. The protein levels of SAT1 expression in normal brain tissues and gliomas were examined using the online tool of Human Protein Atlas (HPA) (www.proteinatlas.org).

### Survival analysis

Kaplan–Meier estimation and a Cox regression model were used to calculate the association between SAT1 mRNA expression levels and OS rate through the "survival" package in R-language. For the Kaplan–Meier survival plot, we defined the low and high expression groups with the median SAT1 expression as the cutoff value. For the Cox analysis, the SAT1 was assessed as a continuous variable. When the *p* value was less than 0.05, two-group survival curve differences were considered as significant difference.

### Association of tumor mutational burden (TMB) with SAT1 gene expression levels

The TMB is the sum number of somatic gene coding errors, deletions or insertions, and base substitutions detected in per million bases. The somatic mutation data were downloaded from UCSC Xena website. We selected the “Masked Somatic Mutation” data in four data files subtypes. We calculated the number of mutation per million bases as the TMB for 33 types of tumors patient. We then further explored the relation between TMB and SAT1 expression levels in the 33 tumor types.

### Estimation of immune and stromal scores

Yoshihara et al. constructed an algorithm to estimate the levels of immune and stromal cells in the tissues of malignant tumors using expression data. The immune/stromal scores were obtained to reflect the presence of immune and stromal cells respectively. The tumor purity of the cancer tissue was calculated by analyzing the gene expression signatures of immune and stromal cells via applying the ESTIMATE algorithm. We obtained the ESTIMATE algorithm complete R script from a website (https://sourceforge.net/projects/estimateproject/). Then, the immune and stromal scores of the 33 cancer types were calculated respectively.

### SAT1 expression is correlated with infiltrating immune cells and immune marker sets

Previous studies have exhibited that immune cell infiltration of the TME is a prognostic factor for the survival rate of patients. Therefore, it is of great significance to explore the relationship between SAT1 expression and immune cell infiltration. The Single Cell Portal (https://singlecell.broadinstitute.org/) was developed to facilitate sharing scientific results, and disseminating data generated from single cell technologies, which provides 340 studies and 12,725,073 cells for researchers. Using "glioma" as the search term, the “Single-cell multi-omics profiling of human gliomas” study contains glial, immune and malignant cell gene expression data. CIBERSORT characterizes each immune cell subtype and accurately quantifies distinct immune cell compositions using a deconvolution algorithm. So, we used a deconvolution algorithm and CIBERSORT computed that the 22 cell types and explored the association between SAT1 expression and immune infiltration. We analyzed that the SAT1 expression was correlated with the abundance of immune cells in the 33 cancer types, including naive B cells, memory B cells, plasma cells, T cells CD8, naive T cells CD4, resting CD4 memory T cells, activated CD4 memory T cells, T cells follicular helper, regulatory T cells (Tregs), gamma delta T cells, resting NK cells, activated NK cells, monocytes, macrophages M0, macrophages M1, macrophages M2, resting dendritic cells, activated dendritic cell, resting mast cells, activated mast cells, eosinophils and neutrophils. In addition, to further study the potentially critical relationship between SAT1 expression and various tumor-infiltrating immune cells, we explored the correlation between SAT1 and the immune gene markers of diverse immune cells.

### The prognostic value of SAT1 mRNA expression in LGG

Kaplan–Meier curves were applied to calculate whether the SAT1 expression was related to the DSS and PFI of patients with LGG. To further evaluate the prognostic impact of SAT1 on patients with LGG, Cox regression models were performed by the R software “survival” package to determine whether SAT1 mRNA expression was a prognostic predictor for LGG patients. A difference with a *P*-value < 0.05 was considered statistically significant. The SAT1 expression and other clinicopathological factors, including age, sex, race, histological grade, IDH mutation and radiation therapy were used as covariates. The AUC of the time-dependent ROC curve for OS plotted to estimate the accuracy of actual observed rates with the predicted survival probability. Time-dependent ROC analyses were conducted by “timeROC” R package. By employing “rms” and “foreign” R packages, we formulated a nomogram consisting of relevant clinical parameters.

### LinkedOmics online website analysis

The LinkedOmics (http://www.linkedomics.org/login.php) is an online platform for analyzing 32 cancer types associated multidimensional dataset [16]. The LinkFinder module was used to explore the differentially expressed genes related to SAT1 in the LGG cohort (*n* = 516). The coexpression of SAT1 was analyzed statistically by Pearson’s correlation coefficient, and the results are graphically shown in the form of heat maps or scatter plots.

### Functional and pathway enrichment analysis of SAT1

KEGG is a knowledge base consisting of biological system-based molecular-level information for the systematic analysis of gene functions and utilities. Gene ontology (GO) includes three aspects: biological processes, molecular function and cellular components. To comprehensively elucidate the potential biological effects of SAT1 gene expression changes, the GO and KEGG analyses of the SAT1 were employed using the R language package “clusterProfler”. Nominal *p* < 0.05 was considered as the cut‐off criterion. The detailed GO and KEGG gene sets can be downloaded from the Broad Institute GSEA (http://www.broadinstitute.org/gsea).

### The chinese glioma genome atlas (CGGA) data portal

The CGGA is a user-friendly web application for data storage and analysis that allows scholars and researchers to probe brain tumor data with over 2,000 samples from Chinese cohorts [17]. These data include the whole-exome sequencing, DNA methylation, mRNA sequencing and microarray, microRNA microarray, and corresponding clinical data. The CGGA is a free data portal and no need to require approval from an ethics committee. For validation, the expression RNA-seq data and matched clinical data of 1018 patients were acquired from the official CGGA web site (http://www.cgga.org.cn/).

### Statistical analysis

Statistical analysis and visualization were performed in R 3.6.0. All the packages used in “R” were listed below: “ggpubr”, “TCGAmutations”, “estimate”, “survival”, “survminer”, “forestplot”, “preprocessCore”, “reshape2”, “RColorBrewer”, “colorspace”, “stringi”, “ggplot2”, “timeROC”, “rms”, “foreign”, “ggExtra”, “org.Hs.eg.db”, “clusterProfiler”, “enrichplot”, “fmsb”. The log-rank test was used in Kaplan–Meier survival analysis. Lasso regression was used to evaluate prognostic model. Statistical significance was indicated in the figures as follows: *P* < 0.05 was considered statistically significant, *p < 0.05, **p < 0.01, ***p < 0.001, ****p <  = 0.0001.

## Results

### The SAT1 mRNA expression levels in pan-cancer

**Fig. 1:**
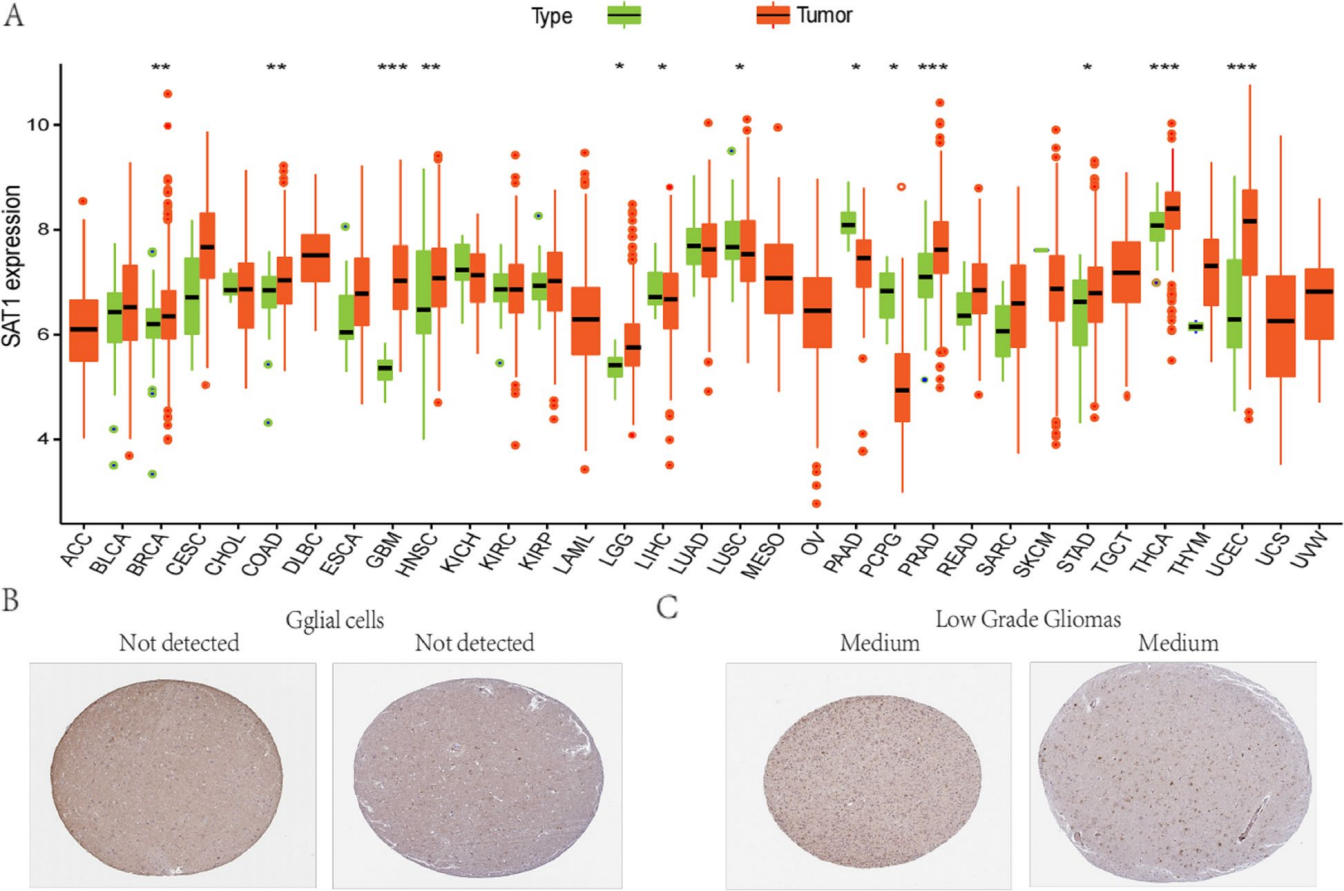
SAT1 expression levels in 33 different types of cancers from TCGA database. Green and orange respectively represent normal and tumor (*P < 0.05, **P < 0.01, ***P < 0.001) (**A**). In normal brain cortex tissues, SAT1 protein was not detectable in glial cells (**B**). LGG cases presented with a medium level of SAT1 staining (**C**). (Adrenocortical carcinoma (ACC), bladder urothelial carcinoma (BLCA), breast invasive carcinoma (BRCA), cervical squamous cell carcinoma and endocervical adenocarcinoma (CESC), cholangiocarcinoma (CHOL), colon adenocarcinoma (COAD), lymphoid neoplasm diffuse large B-cell lymphoma (DLBC), esophageal carcinoma (ESCA), glioblastoma multiform (GBM), head and neck squamous cell carcinoma (HNSC), kidney chromophobe (KICH), kidney renal clear cell carcinoma (KIRC), kidney renal papillary cell carcinoma (KIRP), acute myeloid leukemia (LAML), liver hepatocellular carcinoma (LIHC), lung adenocarcinom (LUAD), lung squamous cell carcinoma (LUSC), mesothelioma (MESO), ovarian serous cystadenocarcinoma (OV), pancreatic adenocarcinoma (PAAD), pheochromocytoma and paraganglioma (PCPG), prostate adenocarcinoma (PRAD), rectum adenocarcinom (READ), sarcoma (SARC), skin cutaneous melanoma (SKCM), stomach adenocarcinoma(STAD), testicular germ cell tumors (TGCT), thyroid carcinoma (THCA), thymoma (THYM), uterine corpus endometrial carcinoma (UCEC), uterine carcinosarcoma (UCS), uveal melanoma (UVW)

To determine the SAT1 expression patterns in different tumor and normal tissues, the mRNA levels of SAT1 in multiple tumor types were analyzed. The analysis showed that compared to adjacent normal groups, SAT1 expression was lower in cancer tissues, including LIHC, LUSC, PAAD, and PCPG. Meanwhile, the expression of SAT1 mRNA was markedly higher in LGG, BRCA, COAD, GBM, HNSC, PRAD, STAD, UCEC, and THCA than in their respective normal tissues (Fig. 1A). We used the HPA to examine the level of SAT1 protein expression in normal brain and glioma tissues. According to the data from HPA, SAT1 protein expression was not detectable in glial cells from normal brain tissues (Fig. 1B). The LGG cases showed medium staining (Fig. 1C). These findings confirmed that SAT1 was expressed at the protein level in glioma tissues and that normal brain had the lower expression.

### Prognostic value of SAT1 mRNA levels in pan-cancer

**Fig. 2:**
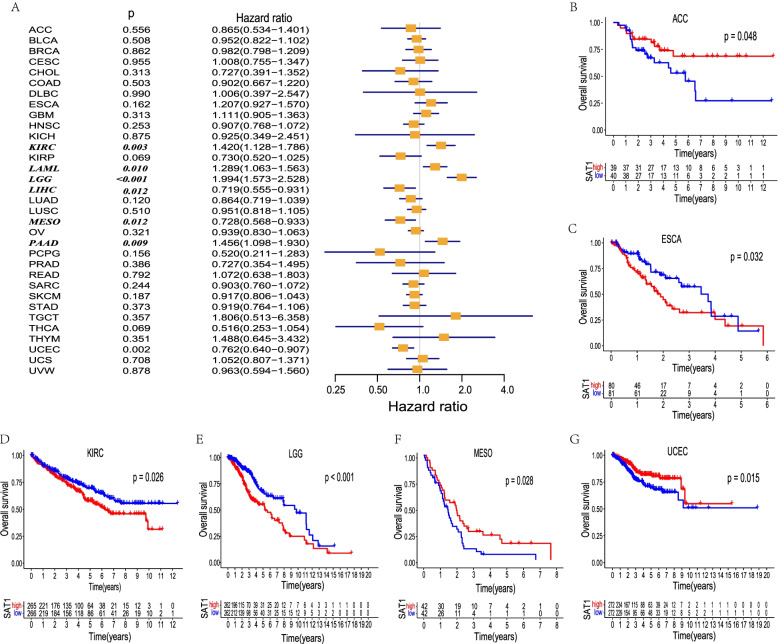
Survival analysis comparing the high and low expression level of SAT1 in different types of cancer in the TCGA dataset. Relation between SAT1 expression and patient prognosis (OS) of different cancers in TCGA database (**A**). The Kaplan–Meier survival curves of OS with significance in six cancer types (ACC, ESCA, KIRC, LGG, MESO, and UCEC) in TCGA (**B**–**G**)

We investigated whether SAT1 expression had prognostic potential for pan-cancer patients. Notably, the expression of SAT1 was correlated with a favorable prognosis in ACC, MESO, and UCEC (Fig. 2B, F, G) using the Kaplan–Meier method. In addition, compared with patients with lower SAT1 expression, patients with high expression SAT1 markedly impacted lower survival in ESCA, KIRC, and especially LGG (Fig. 2C, D, E). Specifically, our analysis revealed that the poor prognosis in KIRC, LAML, LGG, and PAAD was associated with higher SAT1 expression (Fig. 2A) by Cox regression. In contrast, high SAT1 expression was marginally related to favorable prognosis in LIHC, MESO, and UCEC patients. These results corroborated the prognostic potential of SAT1 in some specific cancer types and that increased and decreased SAT1 expression have obviously different prognostic value depending on the different types of tumors.

### Correlation analysis of SAT1 expression and TMB in human cancers

**Fig. 3:**
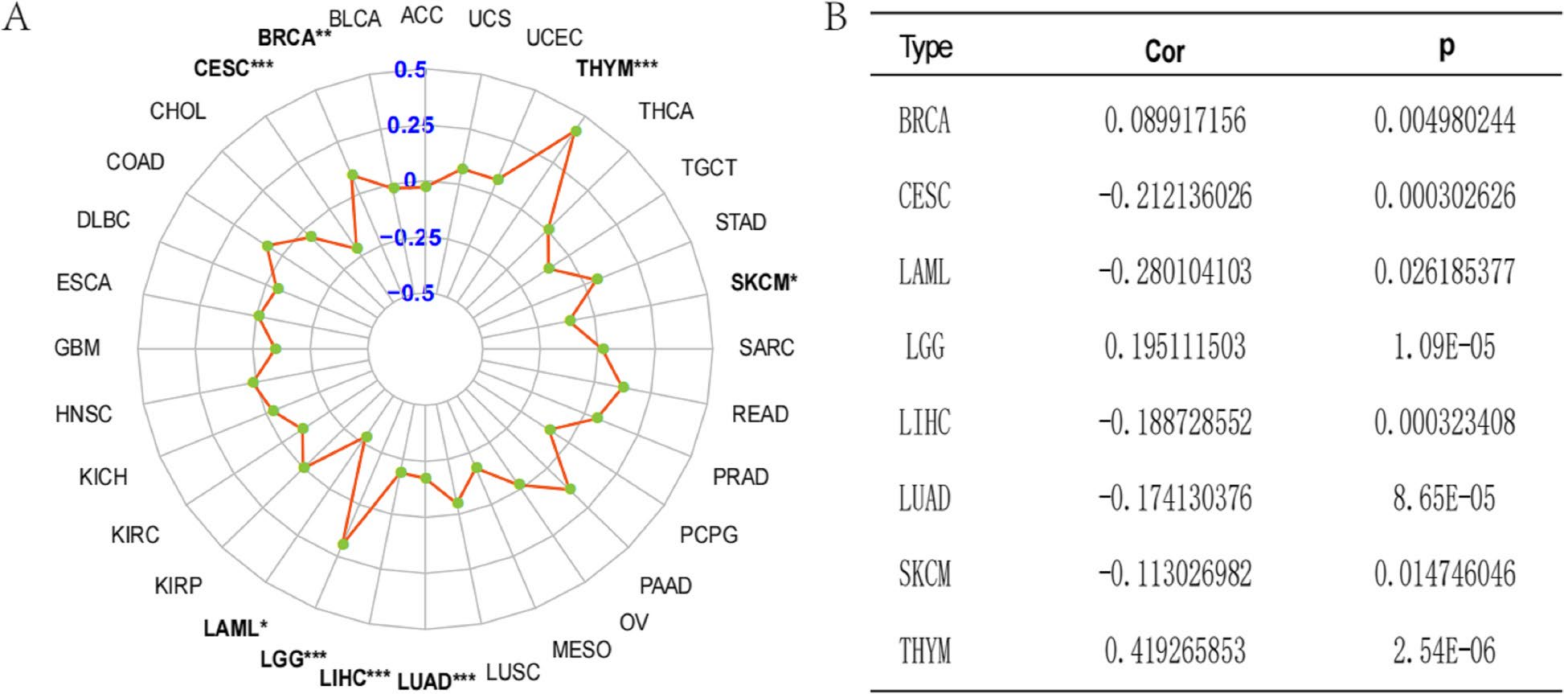
The correlations of SAT1 expression and TMB in cancers, radar map (**A**), detailed correlation coefficient and P value (**B**)

We explored the relationship between SAT1 gene expression levels and TMB in the 33 cancer types using the R software (Supplementary Table S1). Interestingly, the results showed that SAT1 expression was positively related to TMB in LGG, and THYM(p < 0.05 and cor > 0.1) (Fig. 3). Additionally, we also observed that SAT1 expression in CESC, LAML, LIHC, LUAD, and SACM tissues was negatively correlated with TMB. Therefore, the association between the expression of SAT1 gene and TMB is cancer type-dependent.

### Analysis of the relationship between SAT1 expression and various types of immune infiltrating cells and signatures

**Fig. 4:**
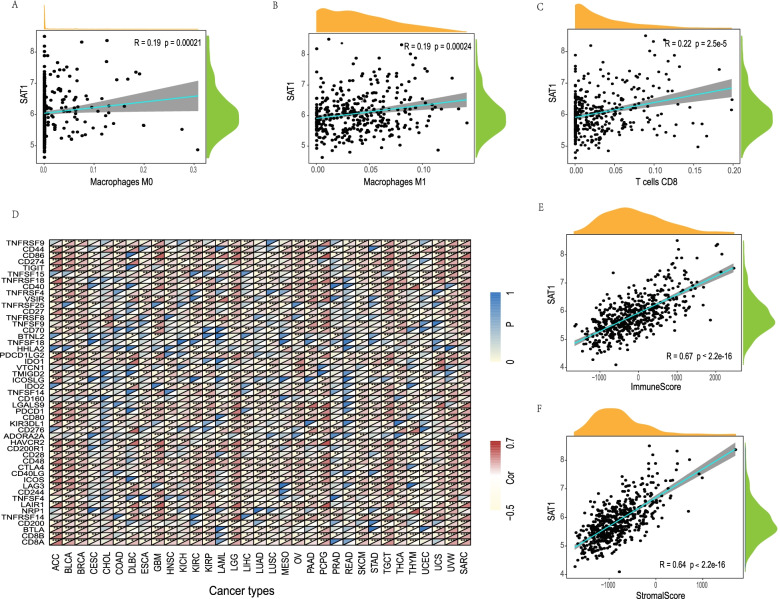
Associations of SAT1 expression to immune cell infiltration in LGG. The correlations of SAT1 expression and immune cell infiltration in LGG (**A**-**C**). Correlation analysis between SAT1 and relate genes and markers of immune cells (**D**). The scatter plots of association between SAT1 and stromal score, immune score in LGG (**E**–**F**). *P* < 0.05 was considered as the difference is of significance

We then used the Single Cell Portal tool to determine that SAT1 was expressed not only on glioma cells, but also on immune cells (Supplementary Fig. S1). It is meaningful to investigate the correlation between infiltration immune cell and the expression levels of SAT1 in different types of tumors. The analysis displayed that SAT1 expression is positively correlated with three types of immune cells, namely M0 macrophages cells (*R* = 0.19, *P* = 0.00021), M1 macrophages cells (*R* = 0.19, *P* = 0.00024), and CD8 + T cells (*R* = 0.22, *P* = 2.5E–05) (Fig. 4A-C). Furthermore, SAT1 expression was also significantly associated with the infiltration levels of many kinds of immune cells in 32 cancer types, respectively (Supplementary Table S2 1–2). To further investigate the correlation between SAT1 and the diverse infiltrating immune cell levels, we focused on the relationship between SAT1 and various immune markers. The results directly denoted that the SAT1 expression level was markedly related to 44 out of 49 immune marker sets in LGG (Fig. 4D).

### Association of SAT1 expression with immune and stromal scores

The immune and stromal cores were calculated by using the ESTIMATE algorithm method, distributing between -1755.64 to 4107.38 and -2500.48 to 2451.06, respectively. The stromal and immune scores have different distributions in different tumor types (Supplementary Table S3). To assess the association between SAT1 and immune and stromal scores, we conducted a correlations analysis. Remarkable correlations were observed between stromal/immune scores and the SAT1 expression levels in LGG **(**Fig. 4E, F**)**.

### Univariate and multivariate models of SAT1 in patients with LGG

**Fig. 5:**
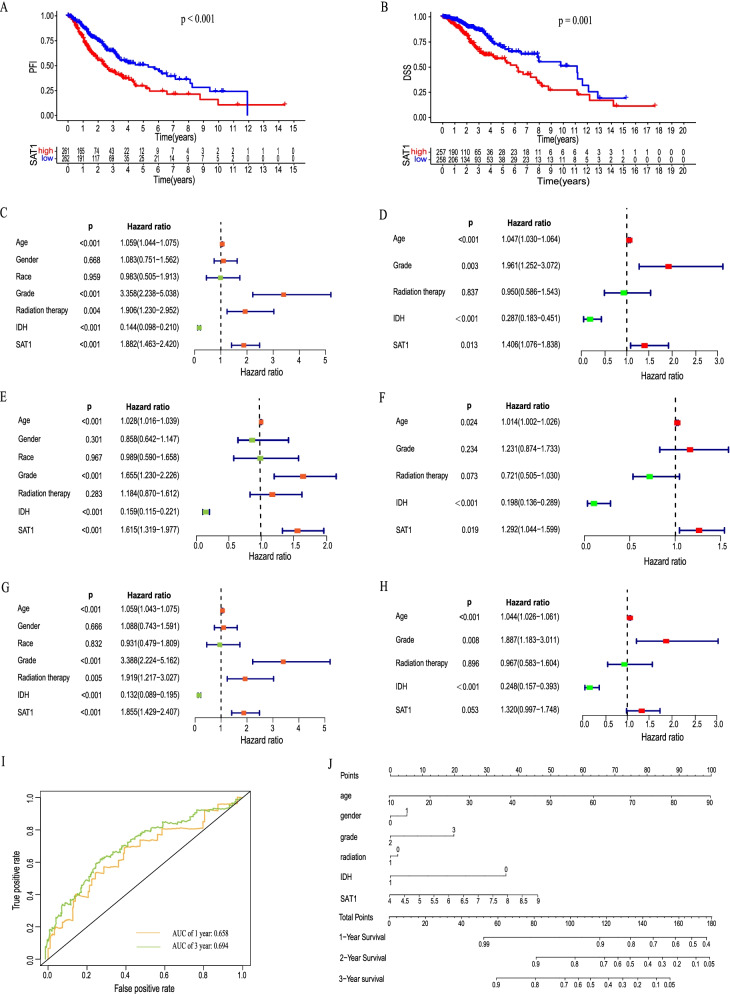
Relation between SAT1 expression and patient prognosis (PFI and DSS) of LGG (**A**-**B**).Univariate and multivariate regression analysis of SAT1 with OS (**C**-**D**), PFI (**E**–**F**), and DSS (**G**-**H**) was performed in LGG patients. Time-dependent ROC for 1-, and 3-year OS predictions (**I**). A nomogram consisting of SAT1 and other clinical indicators for predicting1-, 3-, and 5-year OS of LGG (**J**)

The OS, PFI and DSS of LGG patients with low expression levels of SAT1 were significantly longer than those of patients with high SAT1 mRNA expression levels (all *p* values < 0.05) (Fig. 5A, B). We next performed a univariate regression to verify the relationship between these clinicopathological factors and OS, PFI, and DSS (Fig. 5C-H). The results indicated that age, grade, IDH and SAT1 expression were prognostic factors for the OS and DSS of patients with LGG. Age, grade, radiation therapy IDH and SAT1 expression were significantly correlated with the PFI of patients with LGG. Furthermore, a multivariable model demonstrated that only age, IDH, and SAT1 expression were considered as independent prognostic prediction factors for the PFI and OS of patients with LGG(*P* < 0.05). In conclusion, high LGG expression was independently correlated with shorter PFI and OS in patients with LGG. The area under a time-dependent ROC curve revealed that the 1- and 3- year overall survival of this model was 0.658 and 0.694, indicating the excellent ability to discriminate patients of poor from patients of favored prognosis (Fig. 5I). Next, age, gender, grade, radiotherapy, IDH status and SAT1 were visualized in the nomogram. Nomograms of 1-, 3- or 5-year OS in the cohort are presented in Fig. 5J.

### Associations of differential SAT1 mRNA expression levels with the clinicopathological parameters of LGG patients

**Fig. 6:**
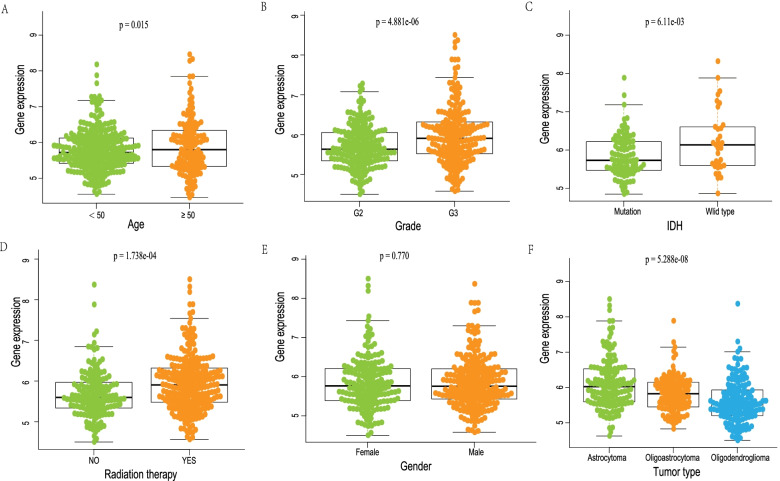
The relationship of SAT1 expression and clinicopathologic parameters in LGG patients, age(**A**), grade(**B**), IDH(**C**), radiation therapy(**D**), gender(**E**), tumor type(**F**)

The correlation between SAT1 mRNA differential expression levels and the corresponding clinicopathological variables was further investigated in LGG patient cohort, including age, sex, histological grade, radiation therapy, IDH, and histological type. As shown in Fig. 6, there were remarkable associations between the differential expression levels of SAT1 mRNA and age (*P* = 0.015), tumor grade (*P* = 4.881e-06), IDH (*P* = 6.11e-03), radiation therapy (*P* = 1.738e-04), and histology type (*P* = 5.288e-08). Conversely, no significant difference in SAT1 mRNA levels was observed with regard to sex (*P* = 0.770).

### Coexpressed genes correlated with SAT1 expression in LGG

**Fig. 7:**
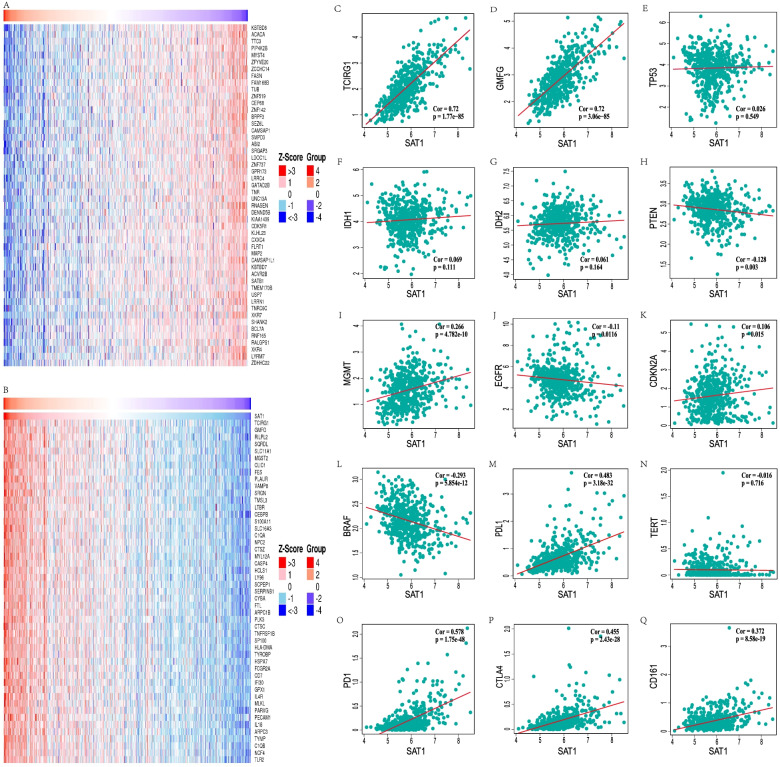
SAT1 co-expression genes in LGG (LinkedOmics) (**A**, **B**). Relationship between interested genes and SAT1 expression level(**C**-**Q**)

To further explore the biological significance of SAT1 in the LGG cohort, we examined mRNA sequencing data by using the LinkFinder function module of LinkedOmics. We found that 6815 genes were absolutely positively correlated with SAT1 expression, while 5454 genes were showed conspicuous negative relationships (FDR < 0.01). A detailed description of coexpressed gene information is shown in the heat map (Fig. 7A, B) and Supplementary volcano map S2. This result indicates that SAT1 has extensive effects on the transcriptome. The expression of SAT1 showed a strong positive correlation with TCIRG1 (*p* = 1.77e-85, *r* = 0.72) and GMFG (*p* = 3.06e-85, *r* = 0.72) expression, which reflects changes in the activation of cAMP-dependent PKA and seemingly directly involved in T-cell activation. IDH1/2 mutation had better response to temozolomide (TMZ) and were associated with the prognosis of glioma patients. MGMT, TERT, EGFR, PTEN, TP53, BRAF, CDKN2A were associated with glioma classification, treatment sensitivity or prognosis. Furthermore, the association between the differential expression levels of SAT1 and interested genes in LGG patients, including IDH, MGMT, TERT, EGFR, PTEN, TP53, BRAF, CDKN2A and immunotherapy related genes (CD161, CTLA4, PD-L1, PD1), was further explored. As shown in the scatter plots in Fig. 7C-Q, there were remarkable correlations between SAT1 expression and IDH2 (*p* = 0.164, *r* = 0.061), MGMT (*p* = 4.78e-10, *r* = 0.226), TERT (*p* = 0.716, *r* = -0.016), EGFR (*p* = 0.0116, *r* = -0.11), PTEN (*p* = 0.003, *r* = -0.128), BRAF (*p* = 5.85e-12, *r* = -0.293), CDKN2A (*p* = 0.015, *r* = 0.106), CD161(*p* = 5.85e-19, *r* = 0.372), CTLA4 (*p* = 2.43e-28, *r* = 0.455), PD-L1 (*p* = 3.18e-32, *r* = 0.483) and PD1(*p* = 1.75e-48, *r* = 0.578).

### GSEA of the SAT1 gene

**Fig. 8:**
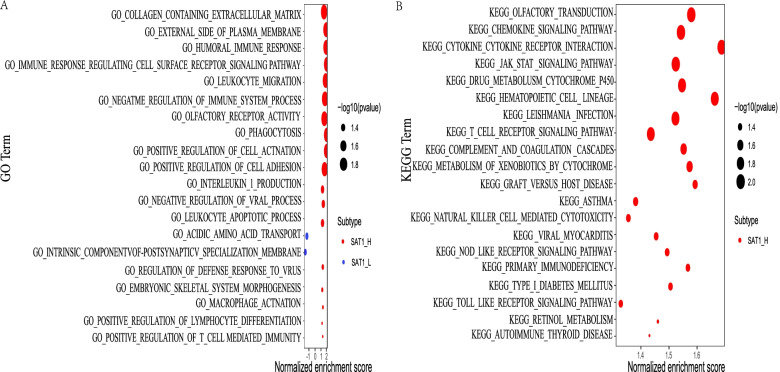
SAT1 gene signature correlated enrichment gene analysis with GSEA. Significantly enriched GO (**A**) annotations and KEGG (**B**) pathways of SAT1 in LGG cohort

GSEA was performed to probe GO and KEGG pathways. Significant analysis of GO terms by GSEA showed that significant enrichment in the HUMORAL IMMUNE RESPONSE (NSE = 1.887), IMMUNE RESPONSE REGULATING CELL SURFACE RECEPTOR SIGNALING PATHWAY (NSE = 1.888), LEUKOCYTE MIGRATION (NSE = 1.729) (Fig. 8A). This suggests that SAT1 may be involved in the immune response processes in LGG. Similarly, the results from KEGG pathways by GSEA showed that SAT1 was closely correlated with the CHEMOKINE SIGNALING PATHWAY (NSE = 1.533), CYTOKINE CYTOKINE RECEPTOR INTERACTION (NSE = 1.675), DRUG METABOLISM CYTOCHROME P450 (NSE = 1.544), JAK STAT SIGNALING PATHWAY (NSE = 1.521) (Fig. 8B). These results indicate that the potential regulatory mechanism of SAT1.

### Further validation of the SAT1 using an independent CGGA cohort

**Fig. 9:**
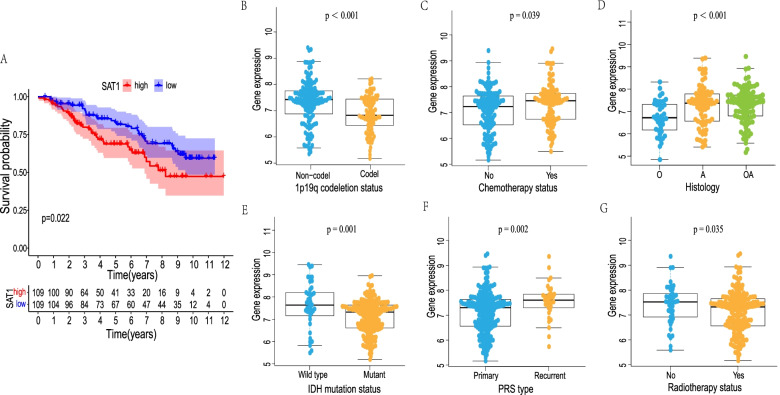
Another independent LGG cohort from the CGGA database was used for further validation. The Kaplan–Meier survival curves of the LGG patients with high and low SAT1 expression level (**A**). The correlation between SAT1 expression and clinical characteristics in LGG patients (**B**-**G**)

Notably, to confirm the prognostic significance of the SAT1 signature in additional LGG cases, another independent cohort from the CGGA database was accordingly validated. A total of 218 LGG patients (cases without relevant data were eliminated) with complete clinical information available were further analyzed. The KM curve analysis exhibited that the LGG patients with high SAT1 expression had significantly shorter OS compared to those with low SAT1 expression (*P* = 0.022) (Fig. 9A). These results implied that the SAT1 signature is reliable and effective for OS prediction across datasets and platforms. Subsequently, we explored the association between the SAT1 signature and clinicopathological parameters (age, sex, histology, radiotherapy, chemotherapy, PRS type, 1p19q codeletion, and IDH mutation status). There were significant differences between SAT1 expression level and radiotherapy (*p* = 0.035), chemotherapy (*p* = 0.039), PRS type (*p* = 0.002), histology (*p* < 0.001), IDH mutation (*p* = 0.001) and 1p19q codeletion (*p* < 0.001) status (Fig. 9B-G).

## Discussion

With the rapid development and wide application of molecular biology techniques, it has been found that many genes can accelerate or reduce the growth and progression of LGG in a variety of ways, including IDH1 mutation, 1p19q codeletion and CDKN2A loss and so on[18, 19]. However, the comparatively great intrinsic heterogeneity in terms of various biological features of LGG in comparison with other tumors restrict the function of individual molecules as markers. Therefore, it is necessary to find more specific and efficacious biomarkers for LGG. To improve prognostic prediction, this study aimed to identify and validate the closely relationship between SAT1 expression and the prognosis of LGG using bioinformatics analysis of the data from two public databases.

Herein we assessed the role of SAT1 expression in 33 different types of cancer using an independent TCGA dataset, indicating notable differences in SAT1 expression between pan-cancer tumor tissues and matched normal tissues. Survival analysis demonstrated that SAT1 expression could be used as a novel prognostic indicator in various cancers, especially in LGG cases. Subsequently, we explored the relation between SAT1 and TMB, immune cell infiltration, and immune cell sets across cancers. It is suggested that SAT1 is highly involved in the process of immune infiltration in various tumors. According to the above comprehensive analysis, we observed that SAT1 was a vital risk factor for LGG. More importantly, the SAT1 was a novel independent prognostic indicator, and abundant SAT1 expression was substantially associated with poorer outcomes in comparison to lower expression. Furthermore, we also performed GSEA to explore the biological pathways and underlying mechanism of SAT1, which laid the solid foundation for further fundamental studies and then proposed possible novel targets for LGG treatment. In addition, the prognostic value of SAT1 was further evaluated with the CGGA database, and the results were consistent with the preceding findings. Otherwise, our analysis demonstrated that SAT1 was closely correlated with IDH status, recurrence, adjuvant radiotherapy and chemotherapy. Ultimately, these results indicated that further investigation of SAT1 expression and its modulation mechanism will be valuable.

Currently, TMZ is the most effective chemotherapy drug used for LGG treatment, and the drug resistance of glioma to TMZ is the crucial reason for chemotherapy failure. Yan et al. confirmed that autophagy plays a vital role in TMZ resistance, and that inhibition of autophagy sensitizes glioma cells to TMZ [20, 21]. Surprisingly, a new death mode, ferroptosis, also plays a major part in the TMZ resistance of gliomas [22]. There is evidence that the activation of the autophagy pathway can trigger ferroptosis, in which inducers accelerate the cell death by inducing the autophagy [23–25]. Chen et al. revealed that ferroptosis inducers (e.g., erastin) can enhance the therapeutic effect of TMZ, which suggests that targeting ferroptosis is a potential mechanism for reversing of TMZ resistance [26]. Importantly, ferroptosis is involved in regulating the sensitivity of rat glioma to radiotherapy (RT). Providing iron-containing water before RT promotes the sensitivity of radiotherapy through the combination of apoptosis and ferroptosis and improves the treatment efficiency[27].

**Fig. 10:**
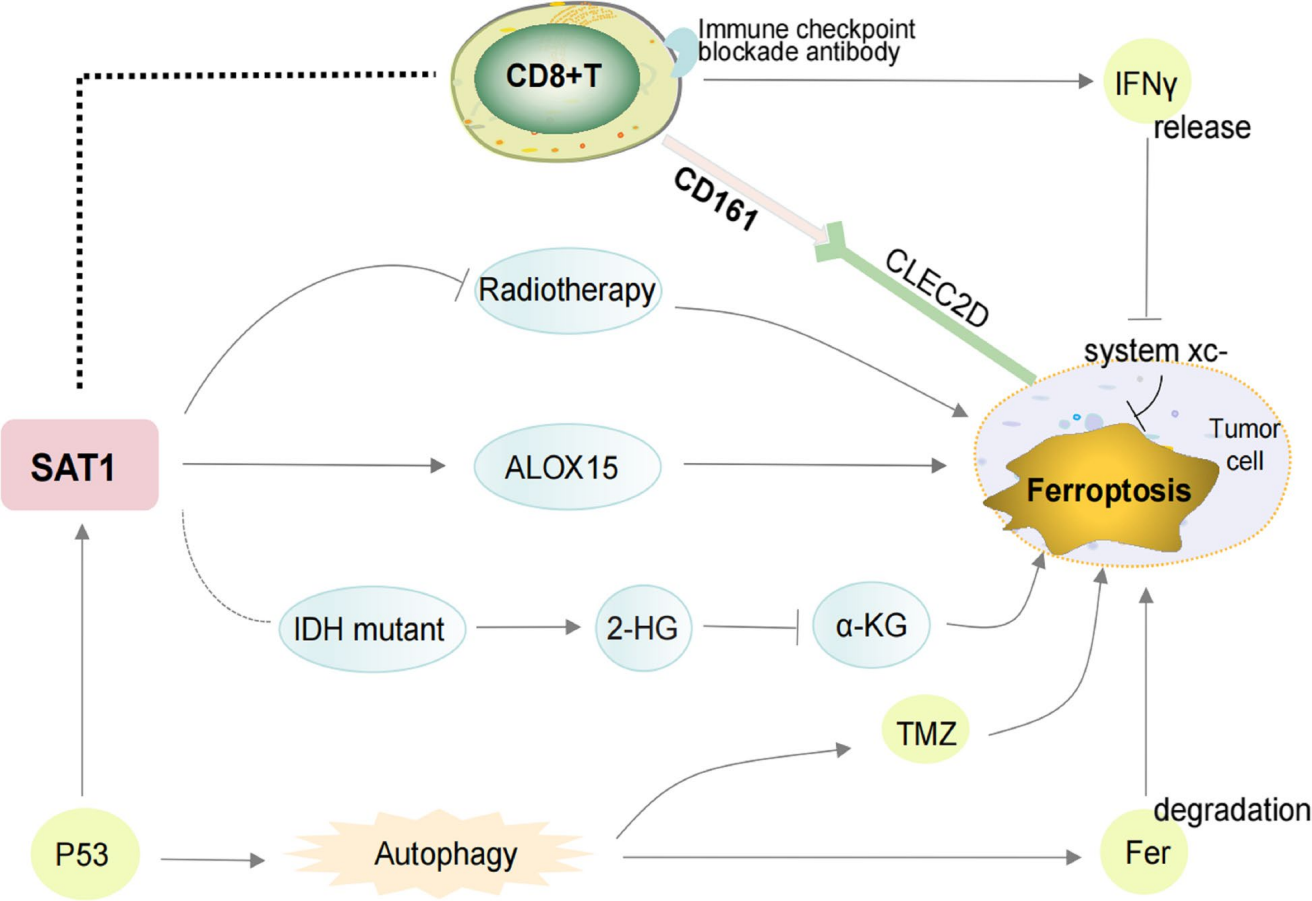
Overview of the relationship between SAT1 and ferroptosis, immune checkpoint blockade and chemoradiotherapy. IDH mutation results in abnormal 2-HG production, which remarkablely suppresses a-KG function, and thus irritate ferroptosis. P53 can promote ferroptosis by enhancing SAT1 which increases the expression of ALOX15. The increased expression of SAT1 was related to the resistance of tumor cells to radiotherapy. The TMZ-driven mechanisms of cell death are dependent of ferroptosis and autophagy in tumor cells. The activation of the autophagy pathway contributes to ferroptosis through the degradation of ferritin. SAT1 is expressed on immune cells and has a strong correlation with CD8 + T cells and its corresponding markers CD8A and CD8B. The immune checkpoint blockade activates CD8 + T cells and potentiates IFNγ signaling pathway to inhibit system xc- expression which leads to induce tumor cell ferroptosis. Tumor-infiltrating cytotoxic T cells expressed the inhibitory NK-cell receptor CD161. Blocking the inhibitory CD161–CLEC2D pathway activated T cell–mediated killing of glioma cells

Recent researchs have revealed that SAT1 activation remarkably induces lipid peroxidation and sensitizes cells to trigger ferroptosis upon ROS production by up-regulating the expression of ALOX15 (Fig. 10) [11]. Interestingly, SAT1-knockout substantially eliminated the ferroptosis induced by p53 and p533KR [9]. This finding further brings new perspectives to the regulation of ferroptosis via polyamine metabolism, and further exploration is necessary to probe the precise role of ferroptosis in the mediation of tumor suppression by SAT1.

In recent years, with the rapid development of molecular pathology, it has been found that several molecular markers (IDH, 1p19q codeletion and MGMT) have an impact on the OS of patients with glioma. Here, we demonstrated that SAT1 was associated with MGMT expression, and reduced IDH mutation and 1p19q codeletion levels. IDH mutation, an essential event in LGG, leads to anomalous production of 2-hydroxyglutarate (2-HG), which obviously inhibits the function of A-KG and promotes ROS accumulation, thus irritating the unique characteristics of ferroptosis [28, 29]**.** Moreover, elevated SAT1 levels in GBM triggered BRCA1 overexpression and drove homologous recombination (HR), subsequently blocking the response of GMB to ionizing radiation (IR) and S-phase drugs, which indicates that SAT1 deficiency is crucial for the efficacy of RT and chemotherapy [6, 30]**.** However, whether SAT1 influences the curative effect of chemoradiotherapy by directly triggering the ferroptosis pathway requires further exploration.

The CNS has traditionally been considered to be an organ of “immunologically privileged” because lacking of lymphatic system and the blood–brain barrier impeded direct contact between the cerebral tissue and the immune system [31]. To our amazement, there is clear and strong evidence that there is still an effective immune response in the CNS. Emerging evidence has demonstrated that the immune microenvironment plays an essential part in malignant glioma, and novel inspiring immunotherapy targeting has great prospects for the clinical management of gliomas. TMB is a novel factor for predicting the effectiveness of immune checkpoint inhibitors, and patients with a lower TMB could have a more unfavorable prognosis if they receive immunotherapy [32, 33]. Intriguingly, our analysis demonstrated that the STA1 gene was closely related to TMB and immune cell infiltration.

Dietrich et al. provided strong evidence to point out that glioma associated microglia/macrophages (GAMs), the major immune components in the tumor microenvironment and consisting of M2 and M1 macrophages that play a role in antitumor responses, are significantly and positively related to cancer grade and unsatisfactory prognosis [34]. Meanwhile, CD8 + T cells, which are celebrated response cells in tumor immunotherapy, are associated with ameliorating prognosis [35]. Compellingly, in this study, we unravelled a highly significant correlation between SAT1 levels and the infiltration of M1 macrophages and CD8 + T cells and their corresponding markers in LGG. The most comprehensive exploration immune checkpoints PD-1, PD-L1 and CTLA-4 are crucial factors that inhibit tumor T cell immunity, and high expression of these molecules had a depressing outcome. It has been recently heralded that CD8 + T cells activated by immune therapy can trigger ferroptosis by inducing lipid peroxidation in tumor cells, and enhanced ferroptosis is conducive to the immunotherapy response [36]. Efimova et al. first proved that ferroptosis is immunogenic in vitro/ vivo, and ATP and HMGB1, the most prominent damage-associated molecules, have been authenticated to be passively released follow the time line of ferroptosis and as immunogenic molecules are tightly related to the immunogenicity of early ferroptotic tumor cells, this implies that ferroptosis is cardinal for the immunotherapy efficacy [37, 38].

In addition, our study shows that SAT1 expression is closely related to CD161 expression. Mathewson et al. inspiringly heralded that tumor-reactive T cells expressed the candidate inhibitory receptor gene KLRB1, encoding the NK cell receptor CD161, which has been certificated to suppress NK cell cytotoxicity by binding to CLEC2D [39]. In addition, that work authenticated that activated T cells mediated glioma cell killing by impeding the inhibitory CD161–CLEC2D pathway, which highlights the CD161 receptor and SAT1 as a novel and potential immunological therapy targets for glioma [40].

The advantages of present study are as follows: First of all, we systematically analyzed the clinical significance and potential biological process of SAT1 in LGG; second, we found that SAT1 is significantly correlated with immune infiltration, which may play an indispensable role in the immunotherapy of LGG patients; and finally, we used CGGA datasets was use to externally verify the prognostic ability of SAT1 and improve the reliability of our results. Nevertheless, a few limitations should be considered. The training and validation datasets may have data heterogeneity, platform differences and sample size differences. Thus, due to the lack of functional verification, and a series of experimental are required to further validate our findings, and prospective efforts should focus on the functional analysis of the roles of SAT1 in inducing ferroptosis and immunotherapy in vivo and in vitro.

## Conclusion

In conclusion, the inspiring breakthrough in glioma therapy by using immune checkpoint inhibitors and ferroptosis inducers has attracted remarkable attention. There is evidence to suggest that the combination of immunotherapy, ferroptosis-targeted therapy, and chemoradiotherapy is potentially valid and practical. Therefore, anticipatory biomarkers are needed to select patients suitable for ferroptosis induction and/or traditional or innovative immunotherapy. Our preliminary outcomes indicated that SAT1 may meet expectations for this distinctive purpose.

## Supplementary Information

**Additional file 1: Table S1. **The relationship between SAT1 gene expression levels and TMB.

**Additional file 2: Table S2. **The correlation between SAT1 gene expression levels and immune cell infiltration.

**Additional file 3: Table S3. **The relationship between SAT1 gene expression levels and immune/stromal score.

**Additional file 4: Figure S1.** The expression of SAT1 in glial, immune and glioma cells.

**Additional file 5: Figure S2.** Co-expression genes correlated with SAT1 expression in LGG.

## Data Availability

The data underlying this study are freely available from UCSC Xena browser (https://xenabrowser.net/), cBioPortal for Cancer Genomics (http://www.cbioportal.or g/) and the official CGGA web site (http://www.cgga.org.cn/). The authors did not have special access privileges.

## Abbreviations

ALOX15: Arachidonate 15-lipoxygenase
CGGA: Chinese Glioma Genome Atlas
CNS: Central nervous system
DSS: Disease-specific survival
GAMs: Glioma associated microglia/macrophages
GO: Gene ontology
GSEA: Gene Set Enrichment Analysis
HR: Homologous recombination
IR: Ionizing radiation
LGG: Low-grade glioma Low-grade glioma
OS: Overall surviva
PFI: Progression-free interval
RT: Radiotherapy
ROS: Reactive oxygen species
SAT1: Spermidine/Spermine N1-acetyltransferase 1
SSAT1: Spermidine/spermine N-1 acetyltransferase
TMB: Tumor mutational burden
