# Supplementary figures and images for: Abundant expression of ferroptosis-related SAT1 is related to unfavorable outcome and immune cell infiltration in low-grade glioma
Source: BMC Cancer. 2022 Feb 28;22:215. doi: 10.1186/s12885-022-09313-w (PMC8883632; doi:10.1186/s12885-022-09313-w)

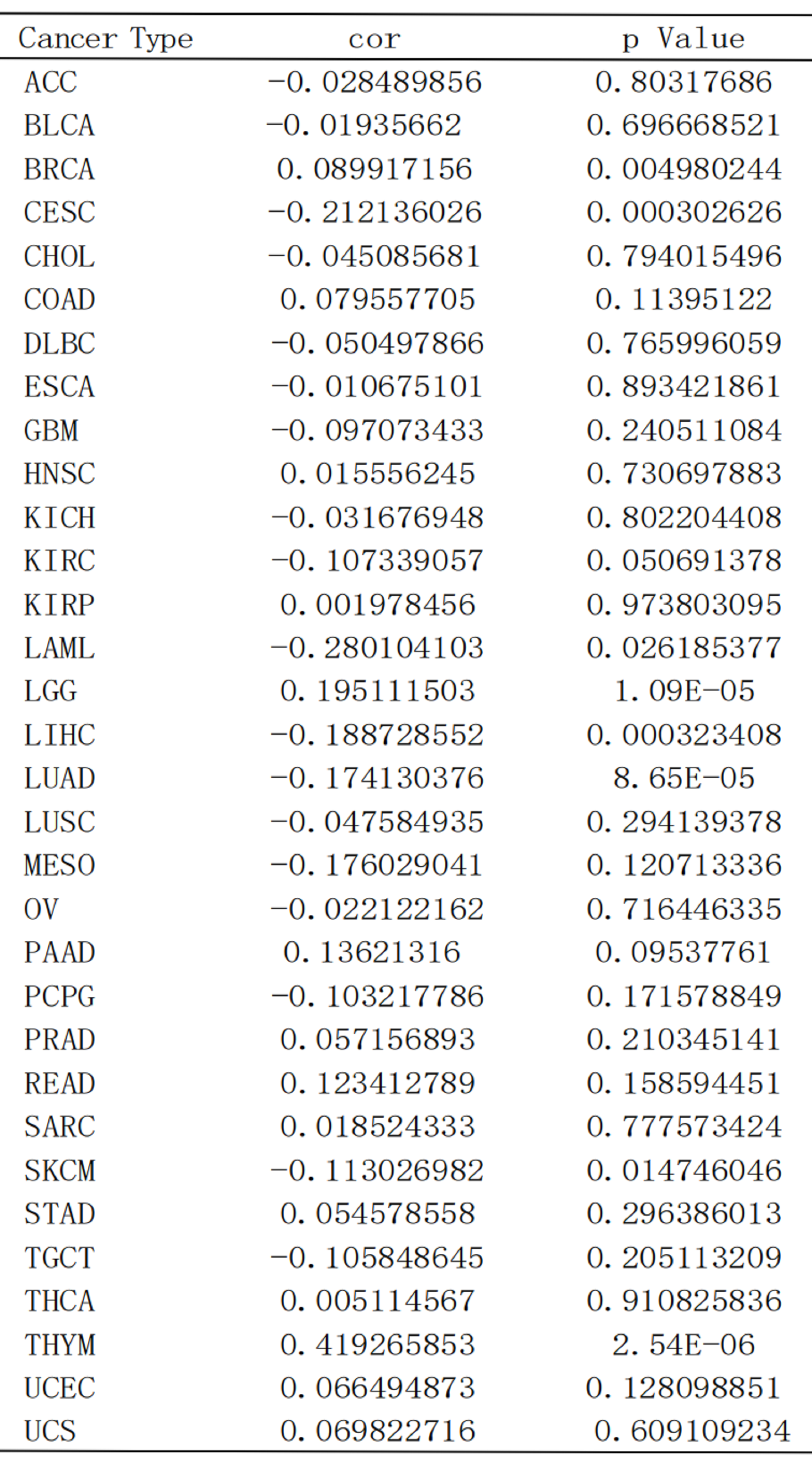

Supplement: Supplementary file 1 — Additional file 1: Table S1. The relationship between SAT1 gene expression levels and TMB. [file 12885_2022_9313_MOESM1_ESM.tif]

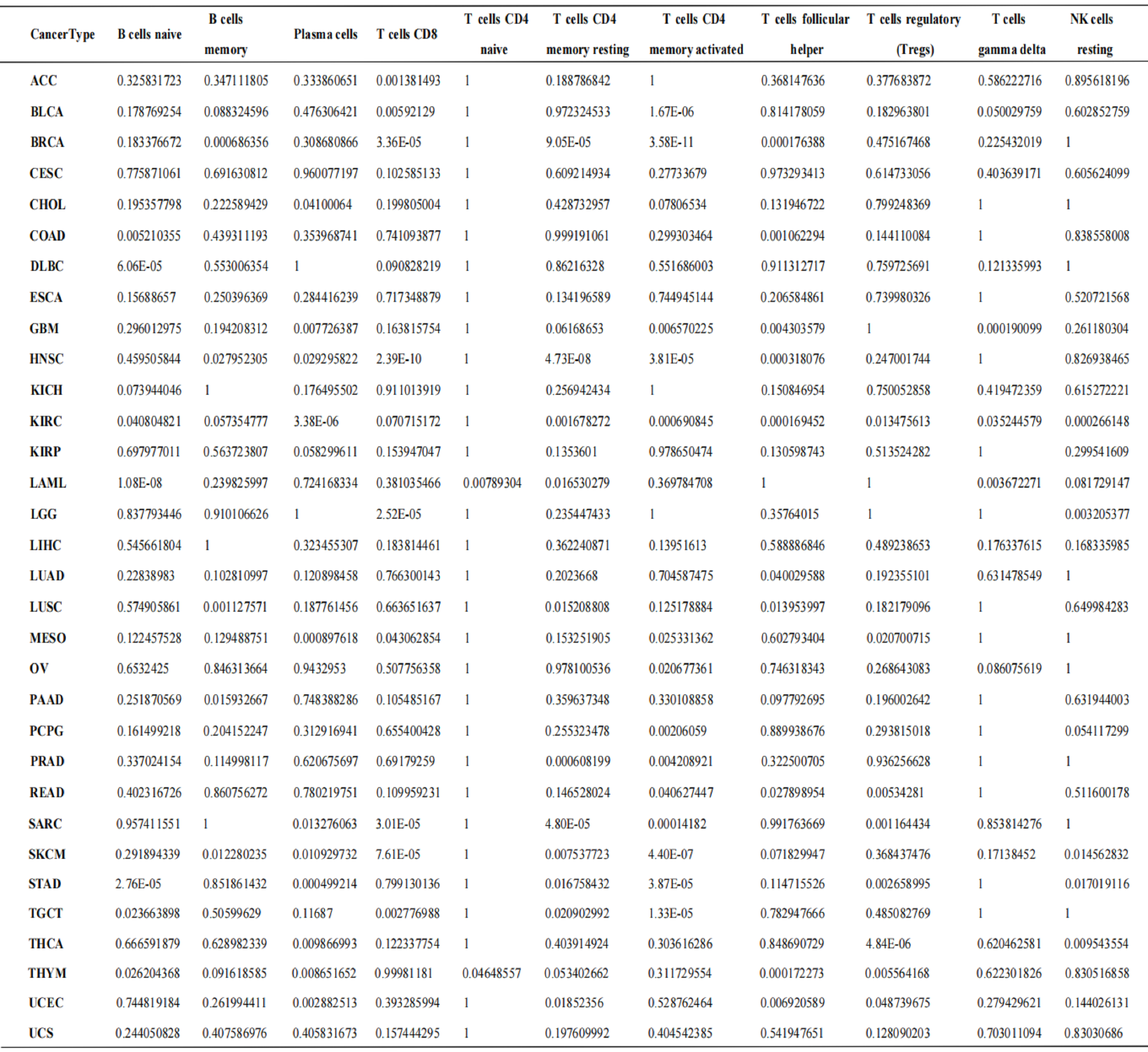

Supplement: Supplementary file 2 — Additional file 2: Table S2. The correlation between SAT1 gene expression levels and immune cell infiltration. [file 12885_2022_9313_MOESM2_ESM.zip › Table S2-1.TIF]

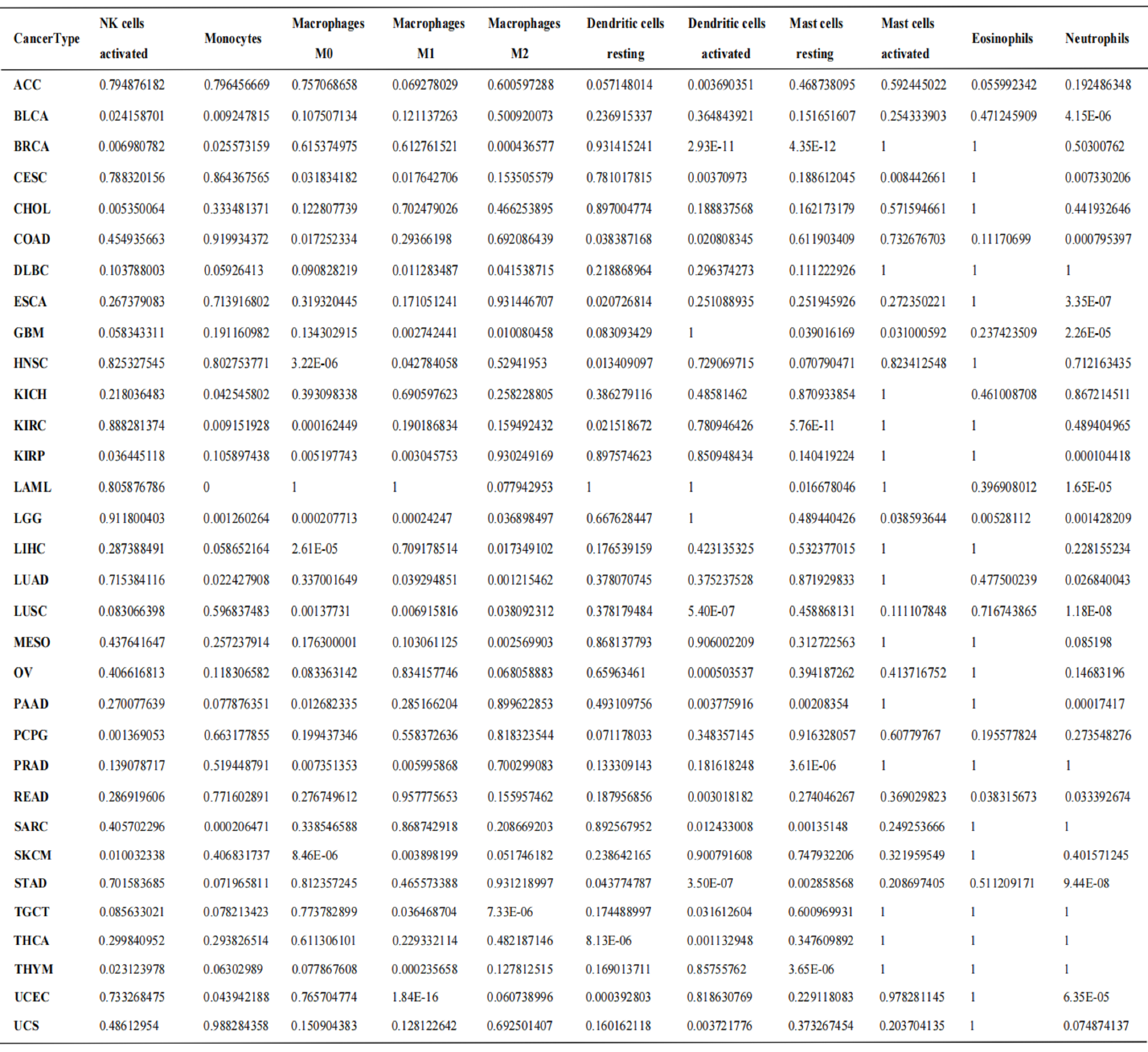

Supplement: Supplementary file 2 — Additional file 2: Table S2. The correlation between SAT1 gene expression levels and immune cell infiltration. [file 12885_2022_9313_MOESM2_ESM.zip › Table S2-2.TIF]

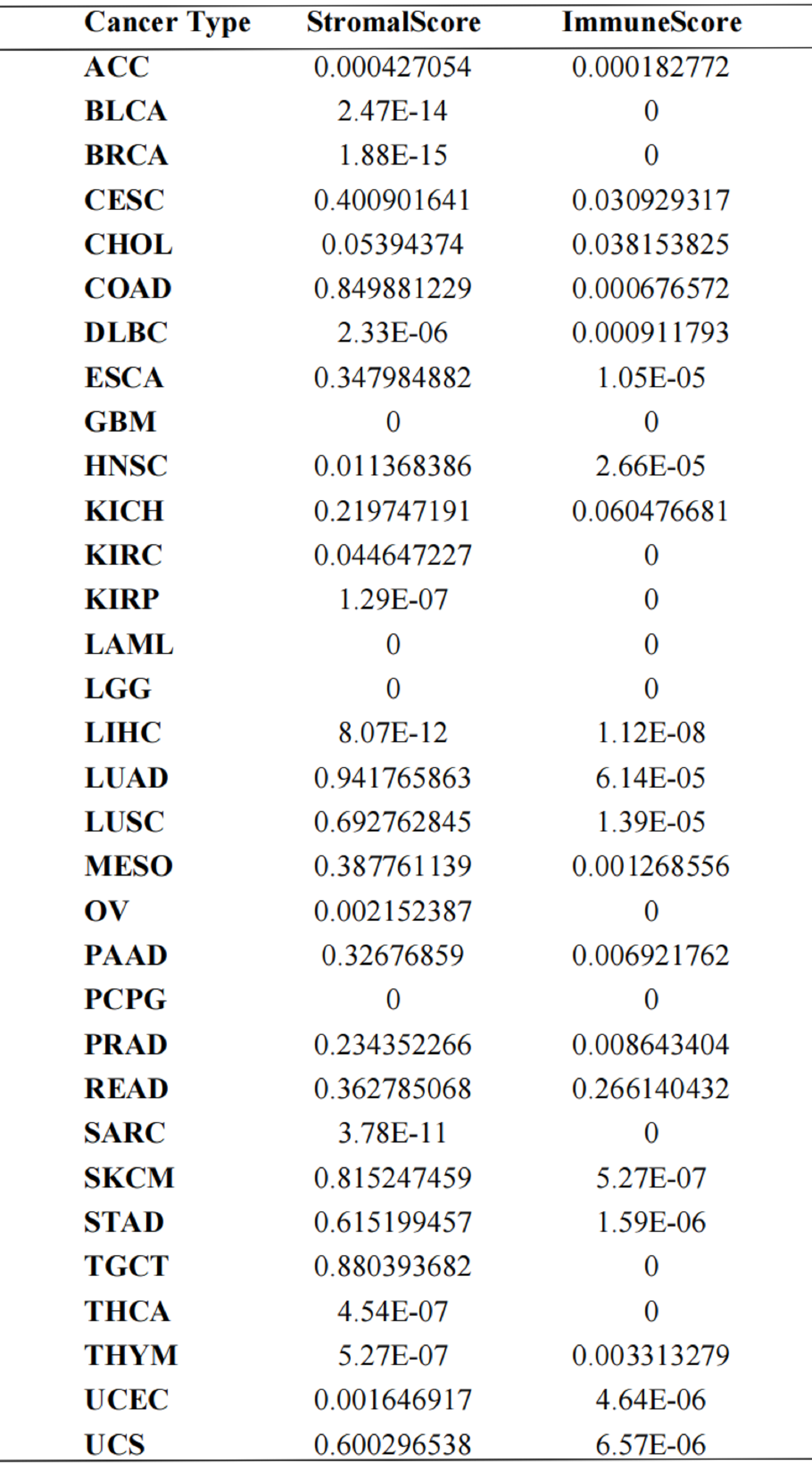

Supplement: Supplementary file 3 — Additional file 3: Table S3. The relationship between SAT1 gene expression levels and immune/stromal score. [file 12885_2022_9313_MOESM3_ESM.tif]

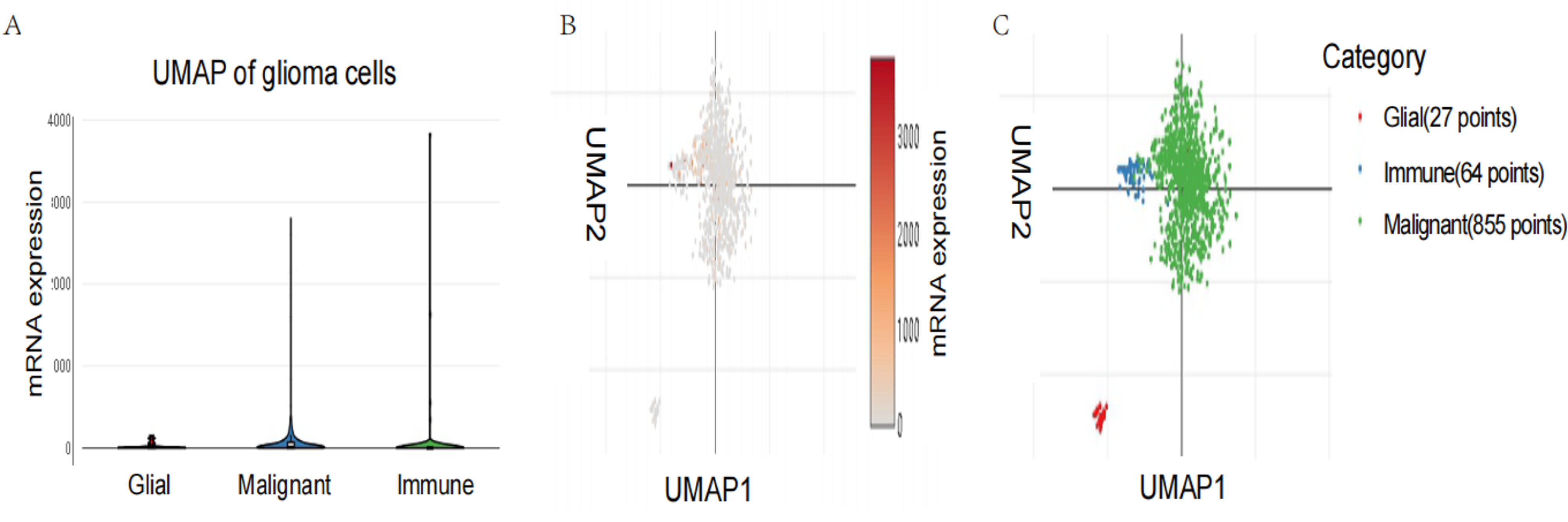

Supplement: Supplementary file 4 — Additional file 4: Figure S1. The expression of SAT1 in glial, immune and glioma cells. [file 12885_2022_9313_MOESM4_ESM.tif]

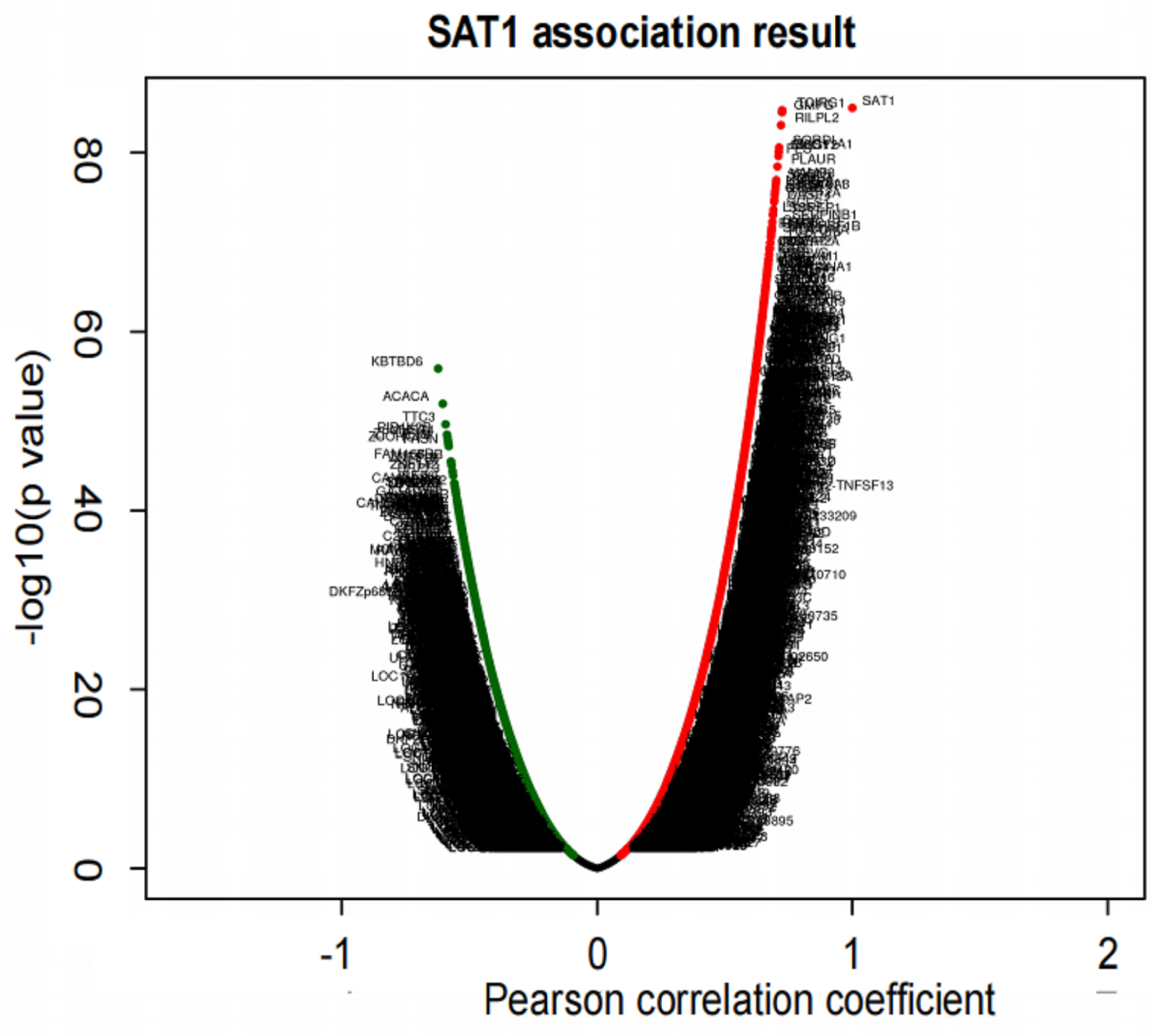

Supplement: Supplementary file 5 — Additional file 5: Figure S2. Co-expression genes correlated with SAT1 expression in LGG. [file 12885_2022_9313_MOESM5_ESM.tif]
